# Urethral Caruncle Presented as Premature Menarche in a 4-Year-Old Girl

**DOI:** 10.1155/2018/3486032

**Published:** 2018-02-13

**Authors:** Manori Gamage, D. Beneragama

**Affiliations:** ^1^Department of Paediatrics, Faculty of Medical Sciences, University of Sri Jayewardenepura, Nugegoda, Sri Lanka; ^2^Department of Pathology, Faculty of Medical Sciences, University of Sri Jayewardenepura, Nugegoda, Sri Lanka

## Abstract

Urethral caruncle (UC) is a benign fleshy outgrowth at the urethral meatus. It was first described by Samuel Sharp in 1750 and occurs mainly at the posterior lip of the urethra, and the exact aetiology is still uncertain. More often it was seen in the postmenopausal women, and only few cases are reported in young girls. Patients may be asymptomatic and could find this as an incidental finding or they may present with symptoms such as dysuria, bleeding per vagina, haematuria, a mass protruding through vagina, and acute retention of urine. Here, we report the case history of a 4-year-old girl presented with vaginal bleeding which was taken as she has attended menarche and found to have urethral caruncle which was the cause for bleeding. Histology confirmed the diagnosis, and girl was completely cured following surgical excision.

## 1. Introduction

In Asian cultures, especially in Sri Lanka, at whatever age a girl gets her first episode of bleeding per vagina parents consider the girl having achieved menarche. As a result, parents hardly question this and seek medical advice. In a young girl who does not have other secondary sexual characteristics suggestive of true menarche, bleeding per vagina could occur due to many reasons. Namely, few of them are trauma, child abuse, congenital hypothyroidism, and foreign body. Here, we report a 4-year-old girl presented to us as premature menarche due to a urethral caruncle.

## 2. Case Report

DS a 4-year-old girl was brought to the paediatric clinic by her mother with a history of significant bleeding per vagina for 4 days duration. She was a healthy girl with no significant past medical history and was not on any regular drug treatment. Mother brought this girl as she had concerns regarding “how her small girl is going to cope with bleeding per vagina monthly at this young age?” There was no history suggestive of exposure to any sexual abuse or trauma to genitals. There was no history of any urinary symptoms.

On examination, she was not pale. She did not have any secondary sexual characteristics to suggest true premature menarche. Her pulse rate was 88 beats per minute, and her blood pressure was 80/50 mmHg. Her abdominal examination was unremarkable. Examination of the genitalia revealed blood stained valva with normal female external genitalia. Her urethral meatus was slightly prominent and erythematous. Her full blood count was normal with a haemoglobin of 11.2 g/dl. Except 15–20 red cells/HPF in her urine analysis rest of the investigations were normal including renal functions.

She was referred to the gynaecologist and was examined under general anaesthesia (EUA). It revealed prominent mucosa at the urethral meatus with continuous oozing of blood. During EUA, the gynaecologist suspected the possibility of urethral caruncle or hyperplastic urethral meatus. This prominent mucosal area was excised and sent for histology. Her postoperative period was uneventful and was discharged home on prophylactic antibiotics.

**Figure 1:**
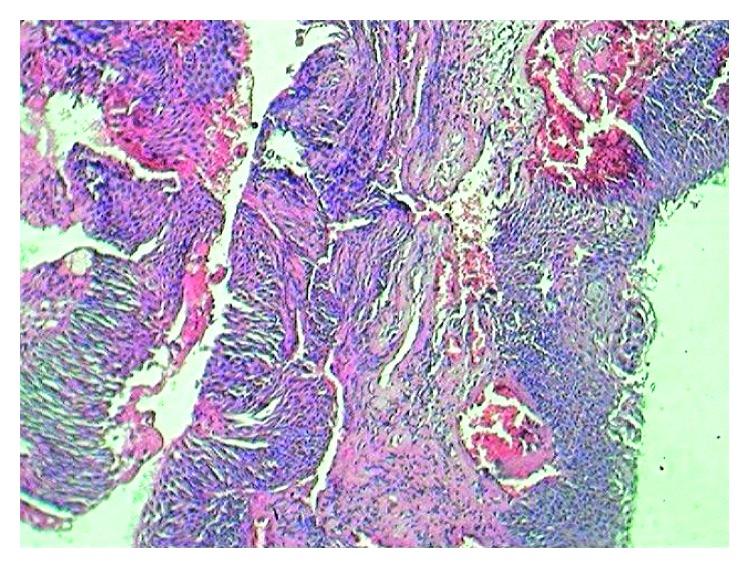
Vascular tissue with transitional epithelium.

**Figure 2:**
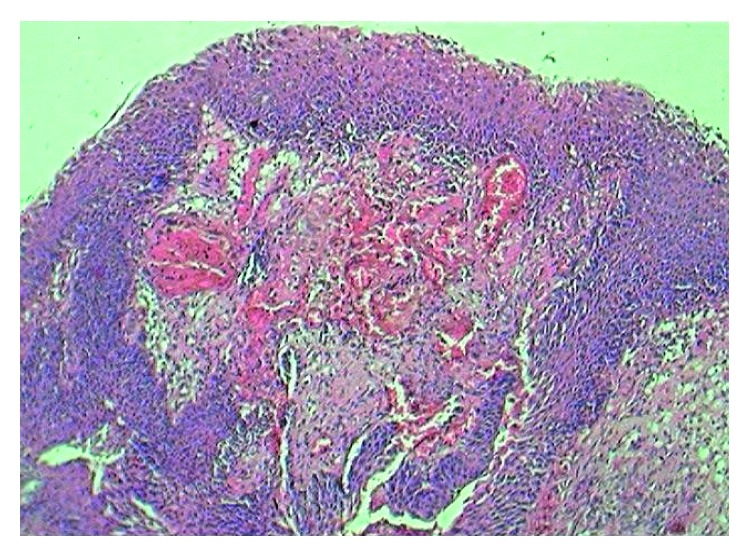
Covering of transitional epithelium.

She was seen in the gynaecology clinic after a week. She was completely well, and there was no recurrence of bleeding per vagina. The biopsy histology confirmed the diagnosis of urethral caruncle (Figures 1 and 2).

## 3. Discussion

Urethral caruncles (UCs) are benign fleshy outgrowths at the urethral meatus [1]. They are known to be the commonest benign tumours found in the female urethra [1, 3]. More often they are seen in the postmenopausal [1] women, and only few cases are reported in young girls [1, 3]. Urethral caruncles are very rare in males [1]. The size may vary from 1-2 mm to 1-2 cm [1].

Urethral caruncle was first described by Samuel Sharp in 1750 [2, 5]. This occurs mainly at the posterior lip [1] of the urethra, and the exact aetiology is still uncertain [3]. But two possibilities suggested as aetiological factors are chronic inflammation and oestrogen deficiency [1, 4]. After analysing few cases reported in children, there was a suggestion of aetiology as congenital [4].

These can be as seen pedunculated or sessile lesions [1]. They are divided in two types according to the clinical features as true caruncles (a vascular papilloma) and pseudocaruncles (a granuloma) [2]. Histology has showed either transitional or squamous cell type as the overlying epithelium.

Patients may be asymptomatic and could find this as an incidental finding or they may present with symptoms such as dysuria, bleeding per vagina, haematuria, a mass protruding through vagina, and acute retention of urine [1]. If it is not visible clearly diagnosis is difficult [4]. When presented as a mass protruding through vagina in young girls, urethral prolapse and malignancy should be excluded [4].

Management options found in the literature are either conservative such as diethylstilbestrol [1, 3] ointment and topical steroid ointments [1, 4] or invasive as surgical excision and electrocoagulation [2]. It is clearly stated that surgical excision is the most preferred method among urologist for larger lesions which are not responding to conservative therapy [6].

## 4. Conclusion

Urethral caruncle is a very rare condition found in prepubertal girls throughout the world. Various forms of treatment have been reported in the literature. But conservative therapy with topical steroid therapy is the core treatment.
